# Erratum for Ricci et al., “Host Traits and Phylogeny Contribute to Shaping Coral-Bacterial Symbioses”

**DOI:** 10.1128/msystems.00917-23

**Published:** 2023-10-11

**Authors:** Francesco Ricci, Kshitij Tandon, Jay R. Black, Kim-Anh Lê Cao, Linda L. Blackall, Heroen Verbruggen

## ERRATUM

Volume 7, no. 2, e00044-22, 2022, https://doi.org/10.1128/msystems.00044-22. Page 11, Table 1, row 3: “9.2” should read “31.5.”

Page 11, Table 1, row 4: “Paragoniastrea australensis” should read “*Paragoniastrea australensis*.”

