# Supplementary figures and images for: Host Traits and Phylogeny Contribute to Shaping Coral-Bacterial Symbioses
Source: mSystems. 2022 Mar 7;7(2):e00044-22. doi: 10.1128/msystems.00044-22 (PMC9045482; doi:10.1128/msystems.00044-22)

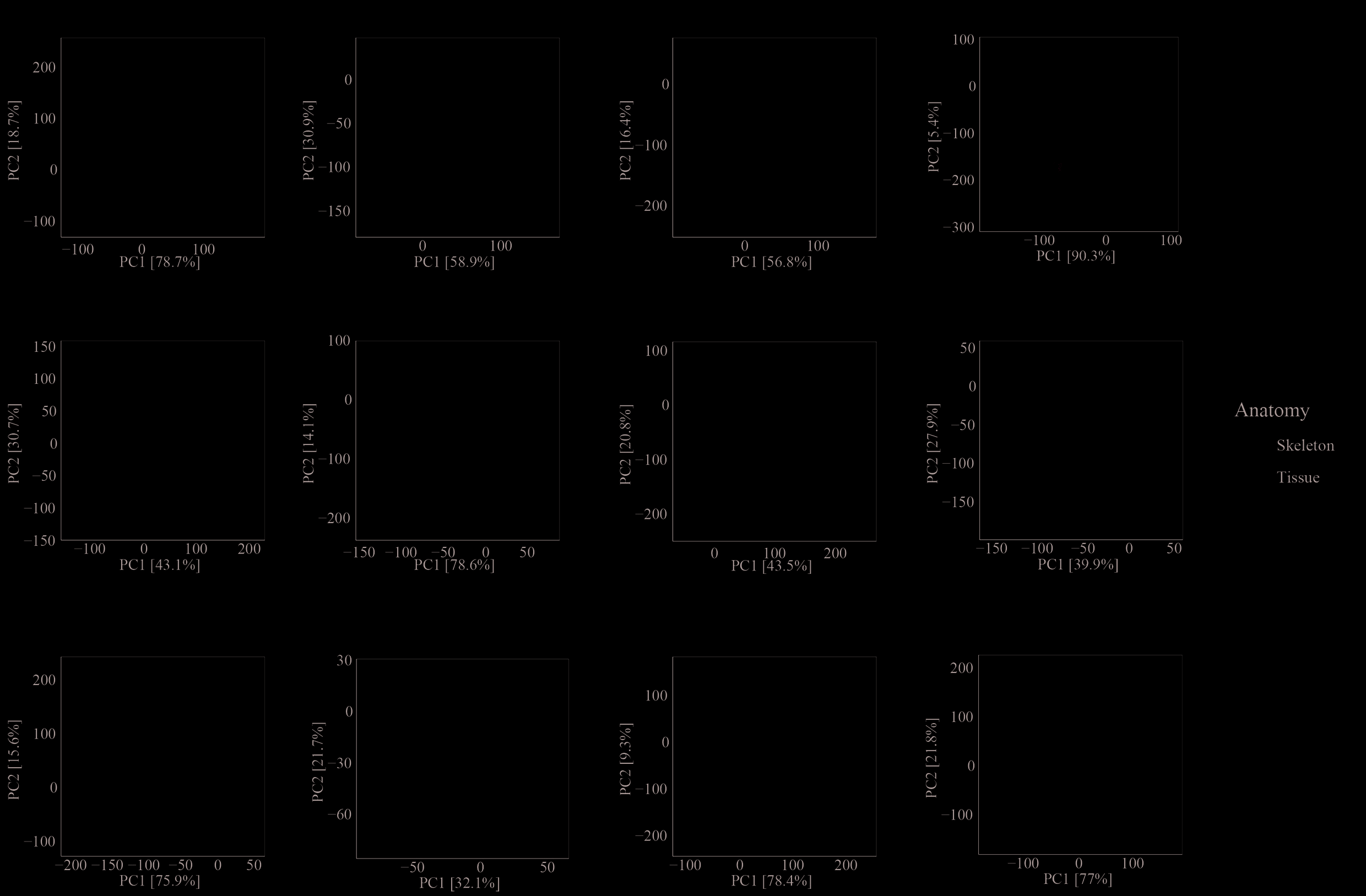

Supplement: FIG S1 [file msystems.00044-22-sf001.tif]

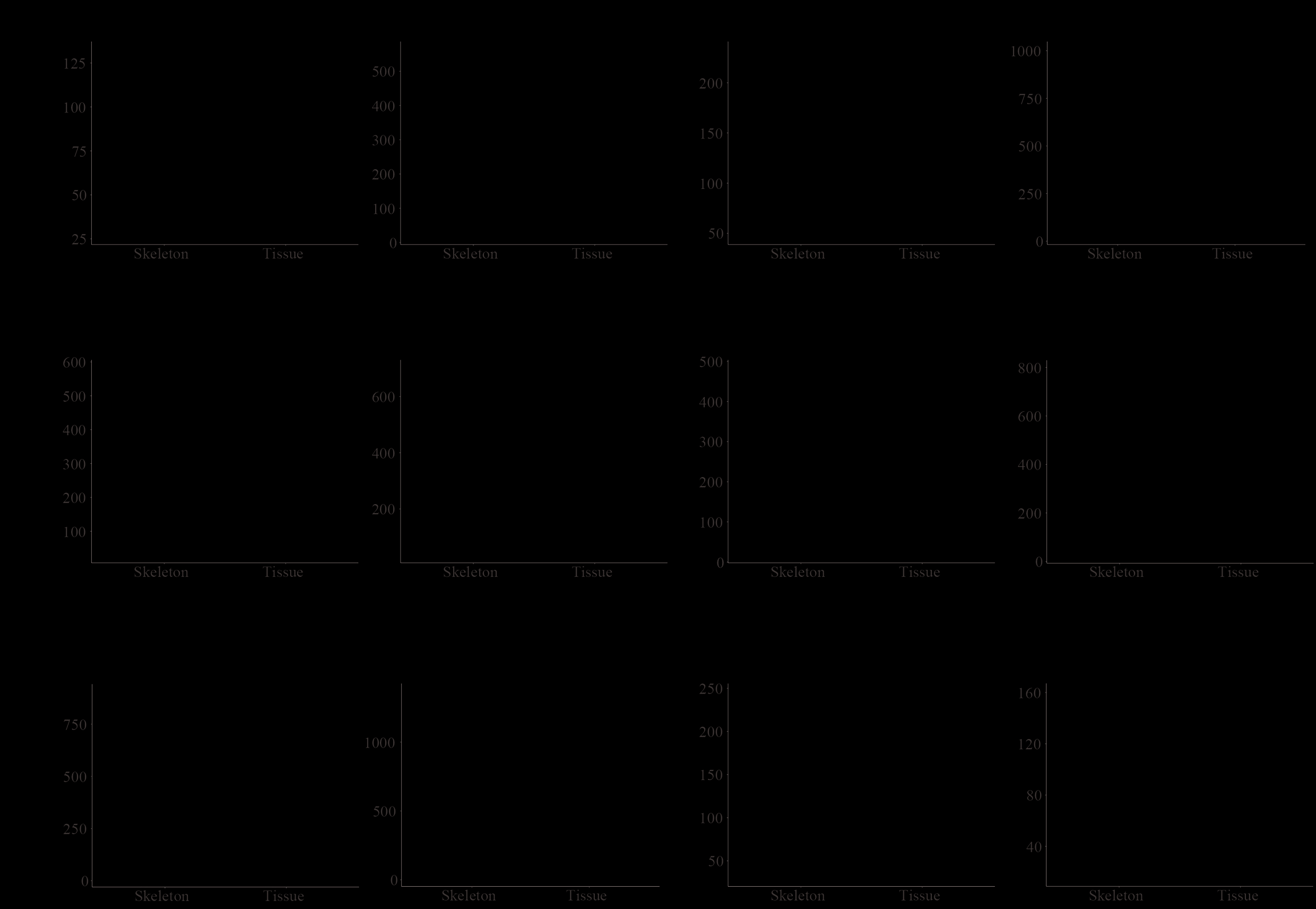

Supplement: FIG S2 [file msystems.00044-22-sf002.tif]

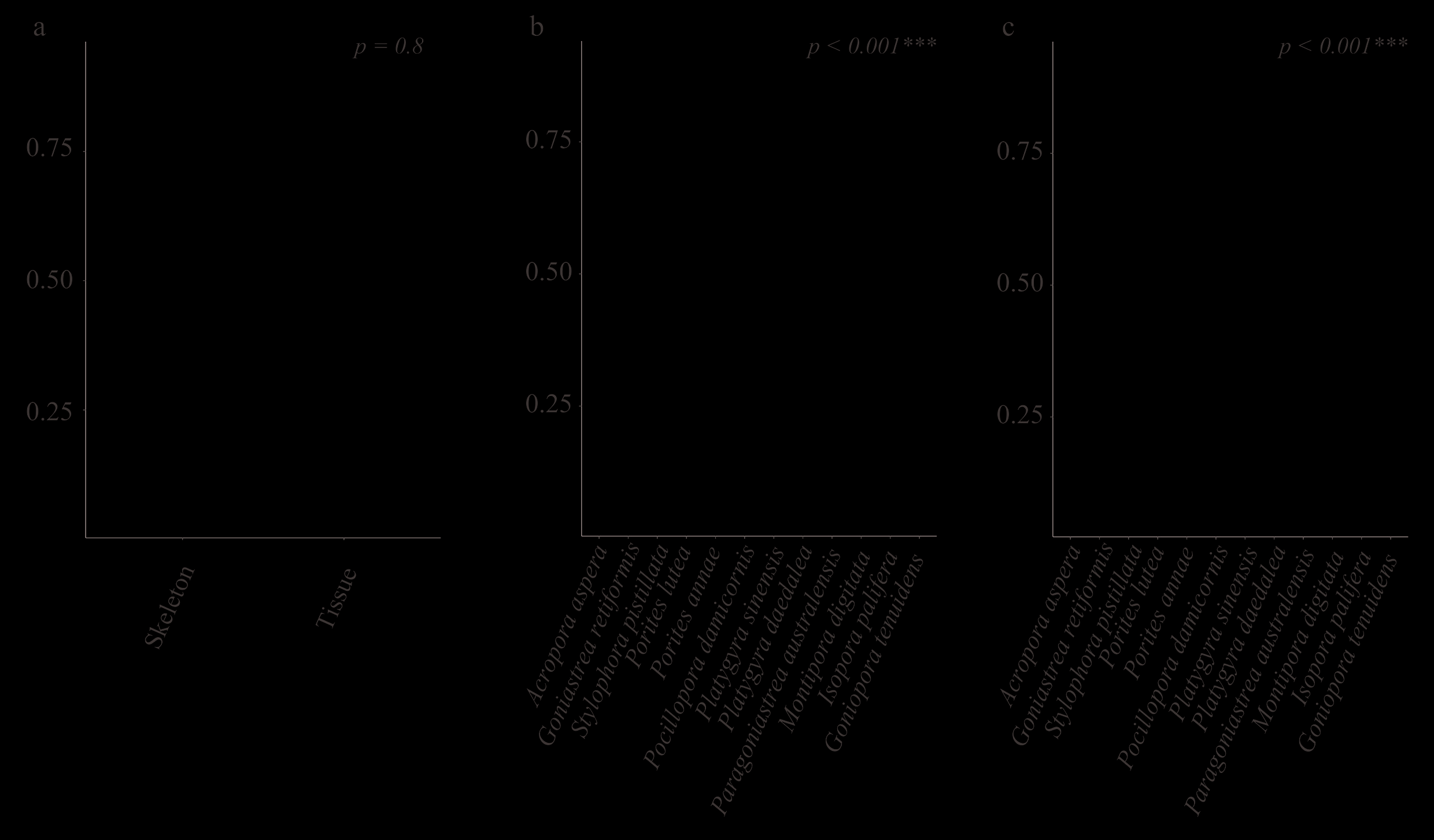

Supplement: FIG S3 [file msystems.00044-22-sf003.tif]

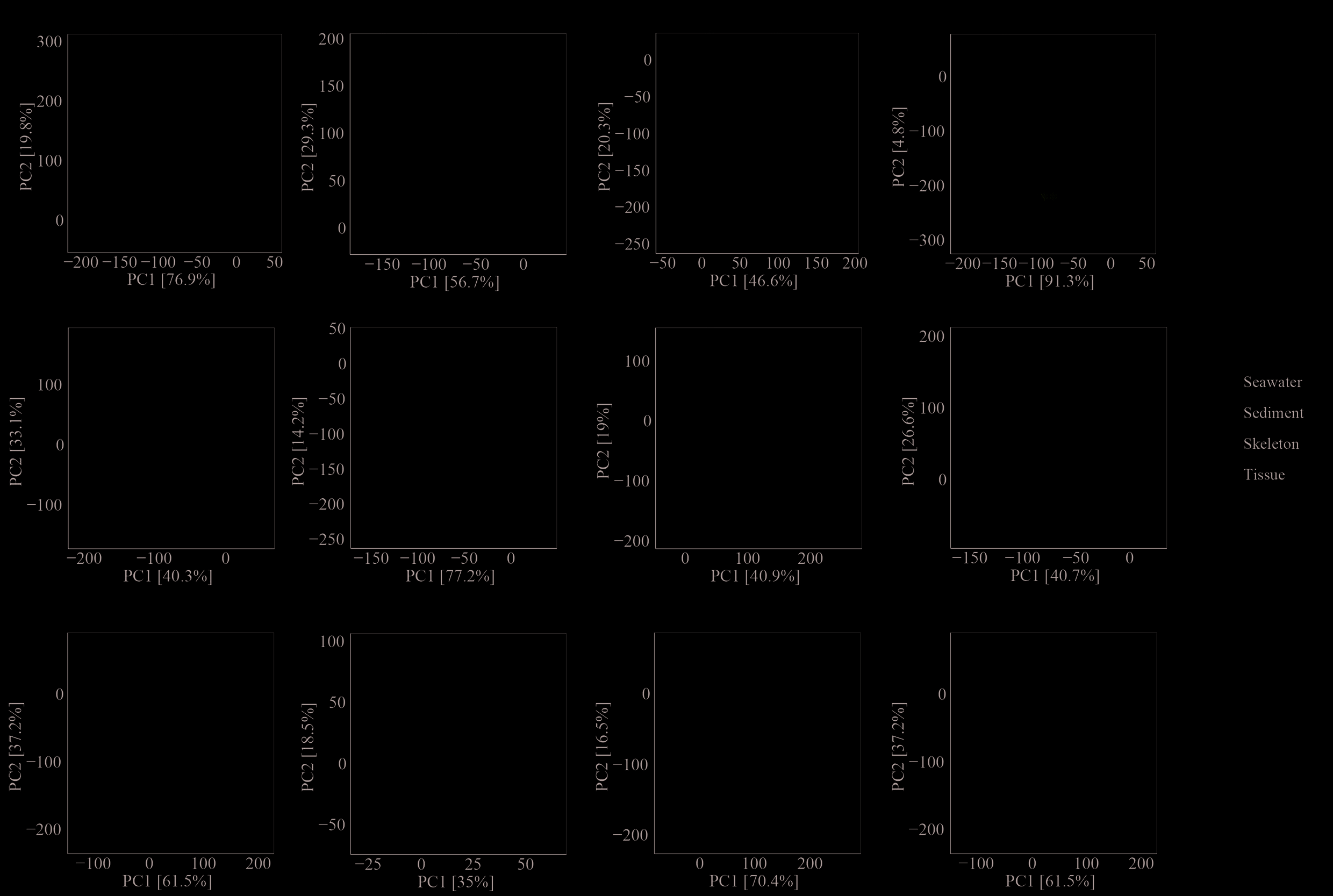

Supplement: FIG S4 [file msystems.00044-22-sf004.tif]
